# The Herbal Pair *Daphniphyllum calycinum*– *Polygonum hydropiper* Alleviates Gastric Mucosal Injury via Regulating Autophagy and Targeting the TLR4/NF‐κB/NLRP3 Pathway

**DOI:** 10.1002/fsn3.71768

**Published:** 2026-04-12

**Authors:** Yunyun Zhi, Keli Zhou, Xueqing Huang, Yonghui Li, Ye Zhu, Yiqiang Xie, Shouzhong Ren

**Affiliations:** ^1^ Engineering Research Center of Tropical Medicine Innovation and Transformation of Ministry of Education, Hainan Provincial Key Laboratory of Research and Development on Tropical Herbs School of Pharmacy, Hainan Medical University Haikou China; ^2^ Department of Traditional Chinese Medicine The First Affiliated Hospital of Hainan Medical University Haikou China; ^3^ Hainan Health Vocational College Haikou China

**Keywords:** autophagy, DCPH, inflammation, M1 polarization, TLR4/NF‐κB/NLRP3

## Abstract

Gastric mucosal injury refers to the pathological changes characterized by congestion, edema, bleeding and erosion of the gastric mucosa caused by various factors. This condition is a significant feature of gastritis in which the inflammatory response plays a critical role in its pathogenesis. The herbal pair *Daphniphyllum calycinum*– *Polygonum hydropiper* (DCPH), known for its anti‐inflammatory and antioxidant properties, has been shown to alleviate gastric mucosal injury. This study aimed to elucidate the mechanism by which DCPH alleviates gastric mucosal injury by integrating network pharmacology with both in vivo and in vitro experimental validation. The results indicated that DCPH treatment significantly increased the antioxidant capacity of the cells and decreased the expression of inflammation‐related factors. DCPH suppressed macrophage M1 polarization while inducing apoptosis in damaged cells. Furthermore, DCPH diminished structural gastric mucosal damage and gastric inflammation. DCPH promotes autophagy by upregulating the expression of LC3II/I and Beclin1, and downregulating P62 accumulation, thereby inhibiting the TLR4/NF‐κB/NLRP3 signaling pathway and M1 polarization, and reducing the release of inflammatory factors. However, these effects can be reversed by the autophagy inhibitor chloroquine (CQ). In conclusion, DCPH alleviates gastric mucosal injury via regulating autophagy and targeting TLR4/NF‐κB/NLRP3, thereby offering insights for the treatment of gastrointestinal diseases.

## Graphical abstract

The schematic diagram illustrated the proposed mechanism of DCPH in mouse macrophages. DCPH promotes autophagy to suppress the TLR4/NF‐κB/NLRP3 pathway and M1 polarization, thereby attenuating cellular oxidative stress and inflammation.

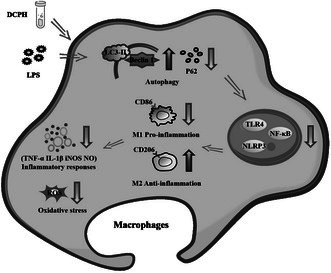

## 1 Introduction

Inflammation is a natural defense mechanism of the body and serves as a protective response to injury. While typically beneficial, prolonged or excessive inflammation can lead to significant harm. Various factors, including physical, biological, chemical, and psychological elements, can trigger inflammation (Ma et al. 2024). When the inflammatory response becomes uncontrolled, it can damage normal tissues and contribute to the development of both acute and chronic inflammatory diseases (Panigrahy et al. 2021; Wu et al. 2025). Research indicates that macrophages, a type of immune cell, play a crucial role in initiating inflammatory responses by releasing inflammatory factors and pro‐inflammatory mediators such as interleukins (ILs), tumor necrosis factor (TNF)‐α, chemokines, ROS, and NO (Gupta and Sarangi 2022). Furthermore, the M1/M2 polarization imbalance exacerbates inflammatory damage to the gastric mucosa. Additionally, autophagy dysfunction leads to the accumulation of damaged organelles and abnormal proteins, intensifying cellular oxidative stress and inflammatory responses. The abnormal activation of the TLR4/NF‐κB/NLRP3 signaling pathway serves as a critical molecular link connecting inflammation, macrophage polarization, and autophagy imbalance. Although traditional non‐steroidal anti‐inflammatory drugs are effective in managing inflammation, they may lead to adverse effects on organs like the kidneys, cardiovascular system, and gastrointestinal tract (Gomes et al. 2025; Ahsan and Sahu 2025). As a result, there is a growing interest in exploring the use of traditional Chinese medicine and natural remedies for the treatment of inflammatory diseases.

Traditional Chinese medicine possesses a wealth of theories and practical experience in treating gastrointestinal diseases. The combination of *Daphniphyllum calycinum* Benth. and *Polygonum hydropiper L*. is a common herb‐pair for managing gastroenteritis in clinical settings. This combination is frequently employed for disorders such as acute and chronic gastroenteritis, gastric ulcer, and diarrhea‐predominant irritable bowel syndrome. The main components of the herbal pair *Daphniphyllum calycinum*– *Polygonum hydropiper* (DCPH), including quercetin, kaempferol, puerarin, and catechins, have been shown to exert their effects by targeting inflammation‐related pathways. Furthermore, studies have demonstrated that autophagy serves as a pivotal degradation pathway for maintaining cellular homeostasis, with DCPH's core components intervening in this process by modulating the expression of autophagy‐associated proteins (Guo et al. 2021). Research has indicated that DCPH exhibits potent anti‐inflammatory, antidiarrheal, antioxidative, and anti‐ulcer properties, demonstrating considerable efficacy in addressing gastrointestinal ailments (Jia et al. 2018; Zhong et al. 2020). However, the precise mechanism of action of DCPH remains unclear, which poses a challenge to its broader application.

The mouse macrophage cell line RAW264.7 induced by LPS serves as a common model for studying inflammation, oxidative stress, and apoptosis (Chen, Peng, and Yu 2020; Braunstein et al. 2025). This study aims to assess the impact of DCPH on LPS‐induced RAW264.7 cells by examining key markers of oxidative stress, inflammation, polarization, and apoptosis. In addition, we further explored the potential molecular mechanism of DCPH's anti‐inflammatory effect and protection of gastric mucosal injury through network pharmacology combined with experiments in vitro and in vivo. This improves the understanding of the mechanism of TCM in treating gastrointestinal diseases and advance DCPH from empirical clinical use to standardized application with well‐defined mechanisms and targets, providing therapeutic options with both efficacy and safety potential for conditions such as acute/chronic gastroenteritis and gastric ulcers.

## 2 Materials and Methods

### 2.1 Reagents

Rapamycin (RAPA) was obtained from Shanghai Aladdin Biochemical Technology Co. Ltd. Chloroquine (CQ) was sourced from Shanghai McLean Biochemical Technology Co. Ltd. The herbal granules of DCPH were acquired from Haikou Pharmaceutical Factory Co. Ltd. in Hainan, China (approval number: National Drug approval number Z10910055, Batch Number: 02230617). DMEM was sourced from Gibco, USA. FBS was obtained from Tianhang Biotechnology Co. Ltd. in Hangzhou, China. Trypsin–EDTA Solution, Penicillin–Streptomycin Solution, Cell Counting Kit‐8, and DAPI Staining Solution were purchased from Biosharp in Hefei, China. Griess reagent NO assay kit, Alexa Fluor 488‐labeled Goat Anti‐Rabbit IgG (H + L), and ROS Assay Kit were sourced from Beyotime Biotechnology in Shanghai. Super ECL Detection Reagent and Annexin V‐FITC/PI Apoptosis Detection Kit were bought from Yisheng Biotechnology Co. Ltd. in Shanghai.

### 2.2 Identification of Active Compounds in DCPH Using UPLC‐MS/MS

The active compounds of DCPH were identified using the ACQUITY ultraperformance liquid chromatography (UPLC) system (Waters, USA), equipped with the ZORBAX Rx‐C8 column (4.6 mm × 5 mm, 1.8 μm, Agilent, USA). The analytes were gradient‐eluted using 0.1% formic acid water (A) and Acetonitrile (B). The elution program is shown in Table 1. The injection volume was 5 μL and the flow rate was 0.3 mL/min. The column temperature was maintained at 40°C. Mass spectrometric (MS) data were detected in positive ion mode based on the electrospray ionization (ESI) source. The ESI settings were as follows: drying gas temperature: 500°C, curtain gas: 25 Psi, collision gas: 10 Psi, ion spray voltage: 4500 V, atomization temperature: 500°C.

**TABLE 1:** Gradient elution program.

Time (min)	A%	B%
0.00	95	5
1.50	95	5
2.50	90	10
14.00	60	40
22.00	5	95
25.00	5	95
26.00	95	5
30.00	95	5

### 2.3 Cell Culture

The RAW264.7 cells, a type of murine mononuclear macrophage, were sourced from the National Collection of Authenticated Cell Cultures in Shanghai, China. These cells were cultured in DMEM supplemented with 10% FBS and 1% penicillin–streptomycin and maintained in a 5% CO_2_ incubator at 37°C. DCPH was initially prepared as a stock solution at a concentration of 20 mg/mL in DMEM, which was subsequently filtered using a 0.22‐μm membrane for further experiments.

### 2.4 Animals and Experimental Treatments

All research involving animals complies with the protocols outlined in the Code of Care and Use of Animals for Scientific Purposes established by the National Health and Medical Research Council of China and has been endorsed by the Ethics Committee at Hainan Medical University. Male KM mice, with weights ranging from 18 to 23 g, were sourced from Hainan Institute of Medicine Co. Ltd. (Haikou, China). They were kept in a clean, ventilated environment with controlled conditions (22°C ± 2°C, 12‐h light/dark cycle) and were given access to water and a standard diet. Before the main experiment commenced, the mice experienced a 5‐day acclimatization period for feeding. The procedures for the animal experiments were conducted in strict compliance with the guidelines for the treatment and utilization of laboratory animals and were approved by the Ethics Committee of Hainan Medical University (HYLL‐2022‐365).

The KM mice were randomly assigned to six groups, with 10 mice in each group. The control group and the model group both received an equivalent volume of distilled water. Mice in the DCPH group and the Omeprazole group were administered DCPH‐L (10 mg/10 g), DCPH‐H (30 mg/10 g), and omeprazole (0.05 mg/10 g) using gavage. Meanwhile, mice in the DCPH‐H + CQ group were given intraperitoneal injections of CQ solution (0.1 mg/10 g) every alternate day, along with daily doses of DCPH‐H. All mice underwent a treatment regimen lasting 7 days. One hour after the last administration, all groups, except the control group, received treatment with anhydrous ethanol (0.15 mL/10 g). Following an additional hour of treatment with anhydrous ethanol, gastric tissue samples were taken, and the damage to the gastric mucosa was evaluated. The collected gastric tissues were then used for histological analysis, real‐time quantitative polymerase chain reaction (RT‐qPCR), and western blotting.

### 2.5 Network Pharmacology Analysis

The chemical components of each herb in the DCPH were sourced from databases such as CNKI, PubMed, and Web of Science. The SMILES notations of the primary chemical components were retrieved from PubChem for target identification in the Swiss Target Prediction database. Genes associated with inflammation, oxidative stress, macrophage polarization, and apoptosis were gathered from GeneCards. A bioinformatics tool was utilized to identify overlapping genes between the primary chemical components and these biological processes. A protein interaction network for the targets was constructed using the STRING online tool. Shared genes were evaluated based on factors like degree centrality, closeness centrality, and betweenness centrality using Cytoscape 3.6.1 software. Furthermore, the Metascape data platform was utilized to investigate biological pathways enriched by these shared targets.

### 2.6 Molecular Docking Simulation

The compound structures were obtained from PubChem and converted to MOL2 format with the help of Open Babel 2.4.1. Additionally, the crystal structure of the protein of interest was retrieved from the RCSB Protein Data Bank. After removing water and ligands, hydrogen atoms were added, charge was calculated, and a new protein in PDBQT format was exported. The size and center of the docking box were determined. AutoDock was used to conduct docking of the active compounds with the target protein individually, selecting the conformation with the highest docking score. Subsequently, the results were analyzed using Pymol and graphical representations were created.

### 2.7 Cell Viability Assays

1 × 10^4^ RAW264.7 cells were seeded in each well in a 96‐well plate. After 24 h, the cells were treated with varying concentrations of DCPH (0, 0.5, 1, 1.25, 2.5, 5, 10, and 20 mg/mL) for 24 h to assess cytotoxicity. Viability of the cells was evaluated utilizing the CCK‐8 Kit.

### 2.8 Detection of ROS

Cultured cells were initially incubated in 6‐well plates for approximately 24 h, then subjected to a 24‐h treatment with DCPH. Subsequently, they were exposed to LPS (1 μg/mL, Solarbio, China) for 12 h to stimulate cellular inflammatory responses. Following this, the cells were incubated in DMEM medium containing the DCFH‐DA probe for 30 min. The levels of ROS were assessed using an inverted epifluorescence microscope (OLYMPUS, Japan).

### 2.9 Detection of NO

Cells were seeded onto 96‐well plates and treated following the standardized procedure. Afterward, 50 μL of cellular supernatant from each category was blended with 50 μL of Griess Reagent I and Griess Reagent II solutions. The optical density at 540 nm for each classification was measured using a multi‐functional enzyme tagging device (BIO‐TEK, USA).

### 2.10 Detection of Apoptosis

Following standard procedures, cells were cultured and treated with the drug before undergoing apoptosis measurement via flow cytometry. The rate of apoptosis was determined using the Annexin V‐FITC/PI Apoptosis Detection Kit as per the manufacturer's instructions. Cells were suspended in 185 μL of binding buffer, stained with 5 μL Annexin V‐FITC and 10 μL PI, and then incubated in the dark at room temperature for 15 min. The stained mixture was filtered through a 300‐mesh nylon filter and analyzed using flow cytometry. Data analysis was performed using NovoExpress v20.1 software.

### 2.11 Evaluation of Macrophage M1 Polarization

After culturing cells and administering medication, flow cytometry was used to assess M1 polarization. The procedure included rinsing cells with PBS, detaching them with trypsin, and centrifuging at 1500 rpm for 5 min at 4°C. Subsequently, 100,000 cells were resuspended in PBS and incubated with an FITC‐conjugated monoclonal antibody against the M1 marker CD86 (0.125 μg, Proteintech, USA). The cells were then resuspended in 400 μL of PBS and analyzed using a flow cytometer.

### 2.12 Immunofluorescence Staining

Cells were cultured in 24‐well plates with cell climbing slices for 24 h. After treatment with DCPH for 24 h, cells were exposed to LPS for 12 h to induce inflammatory responses. The cover glasses were fixed with 4% paraformaldehyde for 20 min, washed with PBS three times, permeabilized with 0.01% Triton‐100 for 15 min, washed again with PBS, and then blocked with 5% BSA for 1.5 h. The primary antibody (1:500 dilution) was incubated with the cells overnight at 4°C. After three PBS washes, cells were incubated with a secondary antibody for 2 h. Immunofluorescence images were then captured and visualized using laser confocal microscopy.

### 2.13 Monodansylcadaverine Staining

Monodansylcadaverine (MDC) staining was utilized as a tracer to detect autophagic vesicles and evaluate autophagy in cell climbing slices. The slices were prepared and processed in batches, following the instructions of the Autophagy/Cytotoxicity Dual Stain Kit (MDC Method) from Solarbio, China (Cat: G0170). The MDC Stain was diluted in a 1:9 ratio with 1 × Wash buffer and applied to the slices, which were then incubated at room temperature for 30 min. Subsequently, the slices underwent three washes with PBS before examination under a fluorescence microscope.

### 2.14 RNA Extraction and RT‐qPCR

Total RNA extraction was performed using the TRNzol Universal Total RNA extraction reagent (TIANGEN, China, Cat: DP424) according to the manufacturer's instructions. Quality and concentration of the extracted RNA were assessed using a multifunctional enzyme label instrument and a micro‐detection plate. Subsequently, cDNA synthesis was carried out in a 20 μL reaction volume, followed by PCR amplification using a real‐time PCR system from Thermo Fisher Scientific (Waltham, MA, USA). The amplification conditions included an initial denaturation at 95°C for 2 min, followed by 40 cycles of denaturation at 95°C for 10 s and annealing at 60°C for 30 s. Relative expression levels of genes were determined using the 2^−ΔΔCt^ method, with the Actin gene as the reference housekeeping gene. Primer sequences for RT‐qPCR are provided in Table 2.

**TABLE 2:** Sequence of primers.

Gene names	Primer‐F (5′–3′)	Primer‐R (5′–3′)
Actin	CCACAGCTGAGAGGGAAATC	AAGGAAGGCTGGAAAAGAGC
iNOS	TTGGGTCTTGTTCACTCCACG	GGCTGAGAACAGCACAAGGG
IL‐1β	TGCCACCTTTTGACAGTGATG	GGAGCCTGTAGTGCAGTTGT
TNF‐α	GTAGCCCACGTCGTAGCAA	GTGAGGAGCACGTAGTCGG
Gpx‐1	GGAGAATGGCAAGAATGAAGA	CCGCAGGAAGGTAAAGAG
SOD‐1	CCATCAGTATGGGGACAATACA	GGTCTCCAACATGCCTCTCT
CD206	ATGCCAAGTGGGAAAATCTG	TGTAGCAGTGGCCTGCATAG

### 2.15 Western Blotting

Proteins were extracted from cultured cells using RIPA buffer (Beyotime, China) supplemented with a protease inhibitor (PMSF, Beyotime, China). The protein concentration was determined with a BCA kit for protein quantification (Biosharp, China). Subsequently, equal amounts of protein samples were separated on a 10% SDS‐PAGE gel and transferred onto PVDF membranes (Millipore, USA). The membranes were blocked with 5% skim milk (Saiguo Biotech, China) overnight at 4°C and incubated with primary antibodies against Bax (Proteintech, USA, 1:8000), Bcl‐2 (Proteintech, USA, 1:4000), NF‐κB p65 (Proteintech, USA, 1:3000), TLR4 (Proteintech, USA, 1:2000), NLRP3 (Beyotime, China, 1:1000), Beclin1 (Proteintech, USA, 1:5000), P62 SQSTM1 (Proteintech, USA, 1:20,000), LC3 (MBL, Japan, 1:1000), and GAPDH (Proteintech, USA, 1:20000). Following three 10‐min washes with TBST, the membranes were incubated with HRP‐conjugated Affinipure Goat Anti‐Rabbit IgG (H + L) antibodies (Proteintech, USA, 1:5000) for 1.5 h. Subsequent to three additional 10‐min washes with TBST, ECL chemiluminescence detection was carried out. Finally, the gray values were quantified using Image J software.

### 2.16 Statistical Analysis

All data are presented as mean ± standard deviation (mean ± SD) from a minimum of three independent experiments. Statistical comparisons between multiple groups were conducted using one‐way analysis of variance (ANOVA). Statistical significance was defined as *p* < 0.05. The statistical analyses were carried out using GraphPad Prism 8 software.

## 3 Results

### 3.1 Chemical Composition of DCPH

**FIGURE 1:**
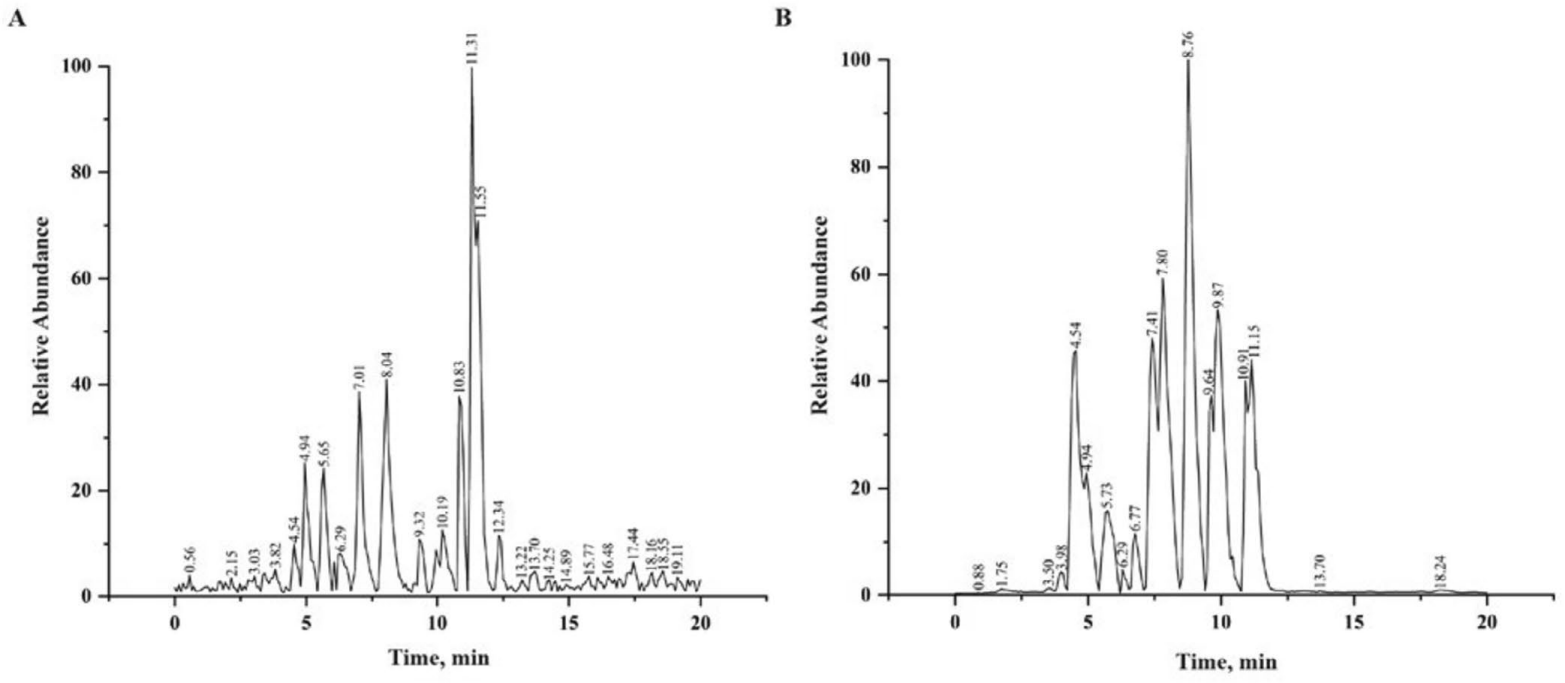
The TIC of (A) DCPH samples and (B) reference substances.

The main components of DCPH were analyzed using UPLC‐MS/MS. By comparing the retention time and the characteristic ion information of each component, 37 main flavonoid compounds were identified (Table 3). The Total Ion Chromatogram (TIC) of DCPH is shown in Figure 1.

**TABLE 3:** The retention time and characteristic ion information of DCPH.

Number	Component name	Molecular formula	Retention time (min)	Ion pair (*m/z*)	Area
1	Dihydromyricetin	C_15_H_12_O_8_	1.04	321.1/321.1	4,312,825.07
2	Taxifolin	C_15_H_12_O_7_	1.06	305.1/61.0	60,827.05
3	Naringin	C_27_H_32_O_14_	1.15	581.2/419.1	76,024.65
4	Daidzein‐8‐C‐glucoside	C_21_H_20_O_9_	1.45	417.1/321.1	46,850.62
5	Phenylalanine	C_9_H_11_NO_2_	1.57	166.0/120.2	1,479,522.46
6	Ferulic acid	C_10_H_10_O_4_	1.86	195.1/81.1	116,369.97
7	Gallocatechin	C_15_H_14_O_7_	1.89	307.0/139.0	848,251.03
8	Catechin	C_15_H_14_O_6_	4.24	291.1/139.0	13,843,892.34
9	Epicatechin	C_15_H_14_O_6_	4.24	291.1/139.0	12,227,967.27
10	Naringenin	C_15_H_12_O_5_	4.85	273.1/69.0	203,039.76
11	Liquiritigenin	C_15_H_12_O_4_	5.46	257.1/137.0	239,070.41
12	trans‐4‐Coumaric acid	C_9_H_8_O_3_	5.99	165.1/92.1	4463.31
13	Formononetin	C_16_H_12_O_4_	7.10	269.1/105.0	26,996.65
14	Rutin	C_27_H_30_O_16_	7.21	611.2/303.0	51,079,038.80
15	Silybin B	C_25_H_22_O_10_	7.78	483.1/153.0	2,336,049.11
16	Diosmin	C_28_H_32_O_15_	7.92	609.2/303.1	95,467.12
17	Luteolin‐7‐glucoside	C_21_H_20_O_11_	8.19	449.1/287.1	15,417,083.35
18	Quercetin 3‐O‐alpha‐rhamnopyranoside	C_21_H_20_O_11_	8.25	449.1/303.1	1,169,947.72
19	Biochanin A	C_16_H_12_O_5_	8.98	285.1/124.0	1,207,899.37
20	Quercetin	C_15_H_9_O_7_	10.39	303.1/153.0	1,342,952.50
21	Luteolin	C_15_H_10_O_6_	10.41	287.1/153.0	6,601,945.13
22	Kaempferol	C_15_H_10_O_6_	10.41	287.1/69.0	888,359.65
23	Daidzin	C_21_H_20_O_9_	10.55	417.1/255.1	1,177,374.88
24	trans‐Ferulic acid	C_10_H_10_O_4_	10.67	195.1/105.0	343,608.76
25	Apigenin	C_15_H_10_O_5_	11.73	271.1/153.0	1,438,035.06
26	Genistein	C_15_H_10_O_5_	11.76	271.1/153.0	2,211,423.05
27	Fisetin	C_15_H_10_O_6_	12.00	287.1/121.0	661,850.15
28	Isorhamnetin	C_16_H_12_O_7_	12.40	317.1/217.1	413,360.46
29	Daidzein	C_15_H_10_O_4_	14.97	255.1/137.0	618,268.89
30	Myricetin	C_15_H_10_O_8_	15.22	319.0/301.0	55,420.31
31	Chrysin	C_15_H_10_O_4_	22.18	255.2/147.0	224,184.34
32	Formononetine	C_16_H_12_O_4_	22.51	269.1/213.1	5048.46
33	Icariin	C_33_H_40_O_15_	23.17	677.2/313.1	272,717.28
34	p‐Coumaric acid	C_9_H_8_O_3_	25.86	165.1/42.0	22,071.26
35	Diosmetin‐7‐O‐rutinoside	C_28_H_32_O_15_	27.30	609.2/286.0	1877.76
36	Isoliquiritigenin	C_15_H_12_O_4_	27.50	257.1/198.0	45,666.42
37	Genistin	C_21_H_20_O_10_	27.51	433.1/215.1	861,165.12

### 3.2 DCPH Exerts Anti‐Oxidantion and Anti‐Inflammatory Effect of RAW264.7 Cells

**FIGURE 2:**
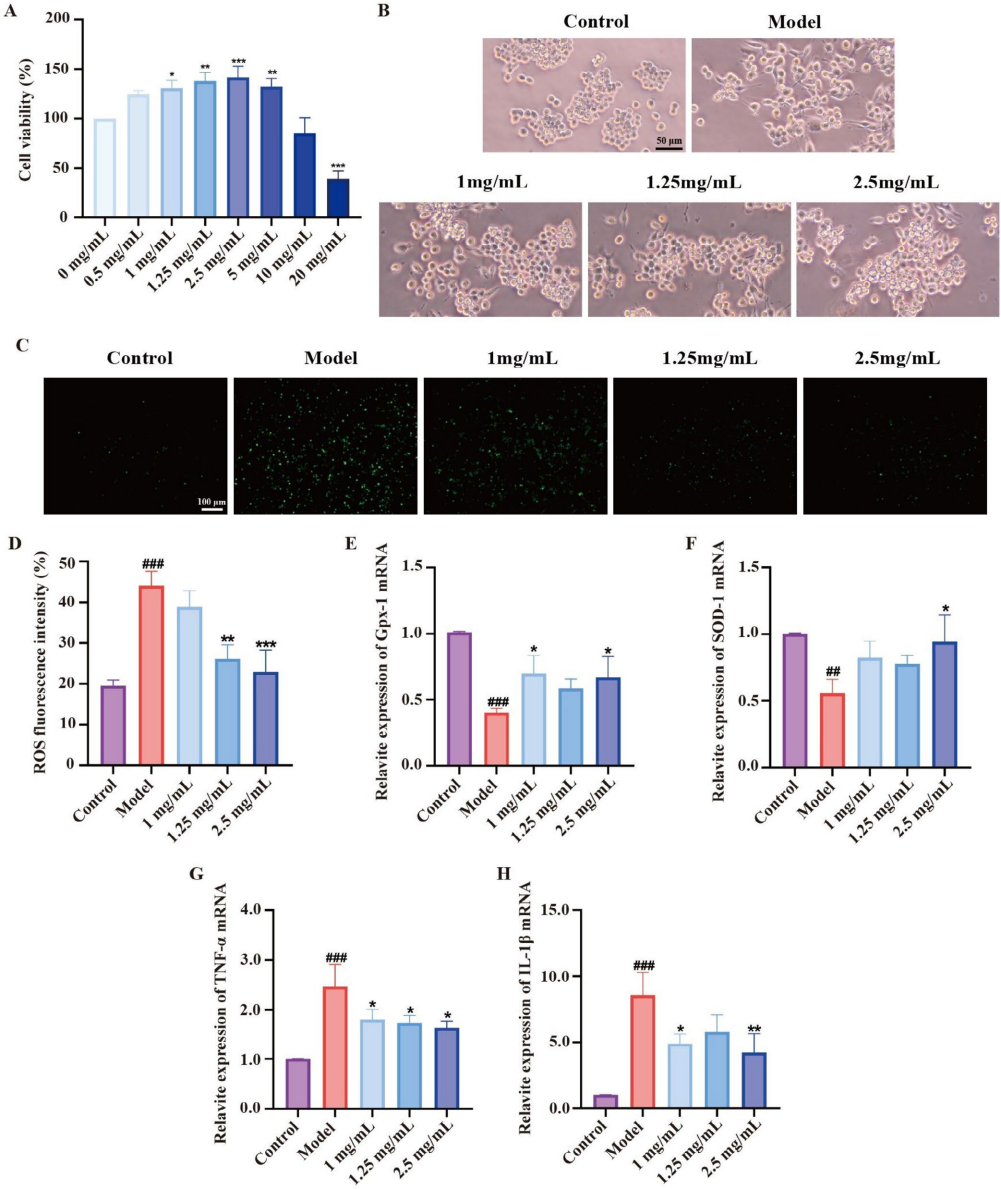
Anti‐oxidant and anti‐inflammatory effect of DCPH in RAW264.7 cells. (A) Cell viability of RAW264.7 cells treated with different concentrations of DCPH for 24 h. (B) Microscopic images of the morphology of RAW264.7 cells treated by DCPH. (C) Visualization of intracellular ROS levels using the fluorescent probe DCFH‐DA. (D) ROS fluorescence intensity. (E) mRNA expression levels of *Gpx‐1*. (F) mRNA expression levels of *SOD‐1*. (G) mRNA expression levels of *TNF‐α*. (H) mRNA expression levels of *IL‐1β*. Each experiment was repeated at least three times. Results are expressed as the mean ± standard deviation. ^##^ *p* < 0.01, ^###^ *p* < 0.001 compared to the control group; *p < 0.05, **p < 0.01, ***p < 0.001 compared to the cModel group.

The cytotoxic effects of DCPH on RAW264.7 cells were evaluated through the CCK‐8 assay. As depicted in Figure 2A, at a concentration of 20 mg/mL, DCPH significantly reduced cell viability, whereas at concentrations of 1, 1.25, and 2.5 mg/mL, it enhanced cell viability in a dose‐dependent manner. Figure 2B demonstrates that LPS induced the polarization of RAW264.7 cells, resulting in the formation of distinct antennae, which is indicative of the M1 phenotype. This polarization was effectively suppressed by various concentrations of DCPH. The antioxidative results showed that DCPH reduced LPS‐induced ROS accumulation in a dose‐dependent manner (Figure 2C,D). Meanwhile, DCPH increased the mRNA expression of Gpx‐1 (Figure 2E) and SOD‐1 (Figure 2F), enzymes involved in ROS clearance, compared to the LPS group. These findings strongly support the antioxidative properties of DCPH.

Additionally, the LPS‐induced RAW264.7 macrophage inflammation model is commonly utilized in vitro to evaluate anti‐inflammatory compounds and investigate inflammatory conditions. Our findings revealed that DCPH significantly decreased the mRNA expression of inflammatory markers TNF‐α (Figure 2G) and IL‐1β (Figure 2H), indicating its anti‐inflammatory properties on RAW264.7 cells.

### 3.3 DCPH Inhibits M1 Polarization and Promotes M2 Polarization in RAW264.7 Cells

**FIGURE 3:**
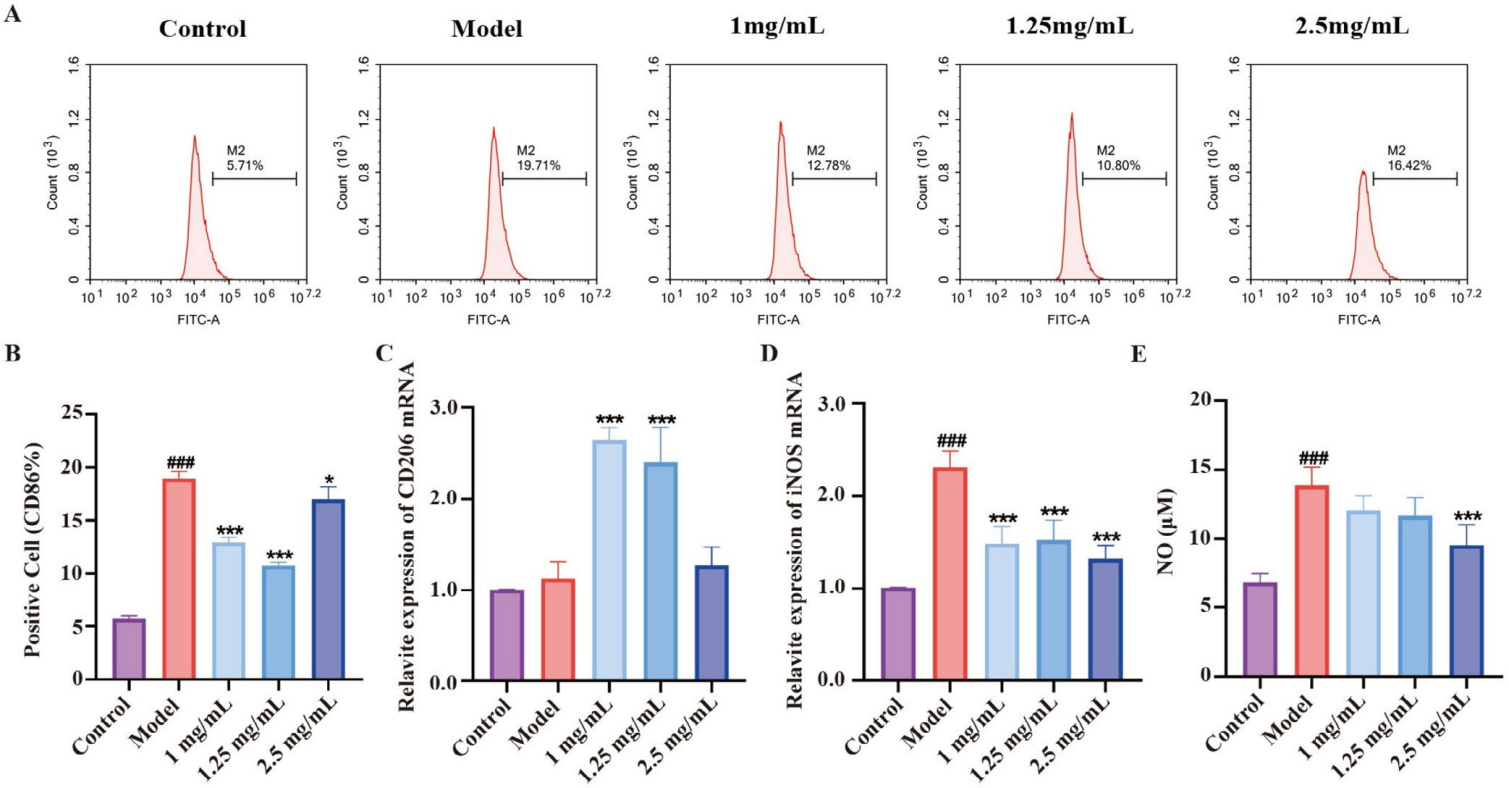
The effect of DCPH on RAW264.7 cell polarization induced by LPS. (A) CD86 expression in RAW264.7 cells was measured by flow cytometry. (B) Percentage of CD86‐positive cells in (A). (C) mRNA expression levels of M2 polarization marker CD206. (D) mRNA expression levels of iNOS. (E) The release of NO. Each experiment was repeated at least three times. Results are expressed as the mean ± standard deviation. ^###^ *p* < 0.001 compared to the Control group; *p < 0.05, ***p < 0.001 compared to the Model group.: O significance.

CD86 and CD206 are membrane surface proteins used as markers for M1/M2 subtype macrophages. In our study, we investigated the effect of DCPH on M1 polarization by analyzing CD86 expression in macrophages through flow cytometry. The results showed a significant increase in CD86 expression in the model group compared to the control group, with the positive expression rate rising from 5.71% to 19.71%. However, this increase was reversed after pretreatment with DCPH (Figure 3A,B). Furthermore, we assessed the mRNA expression of CD206, a marker for M2 cells, using RT‐qPCR. Our results showed that CD206 mRNA expression remained unchanged after LPS treatment. Interestingly, DCPH at concentrations of 1 and 1.25 mg/mL significantly increased CD206 mRNA expression, while no significant change was observed at 2.5 mg/mL (Figure 3C). Additionally, our data in Figure 3D revealed that DCPH effectively reduces the mRNA expression of iNOS, a specific M1 polarization marker, leading to decreased NO levels in the cell supernatant (Figure 3E).

### 3.4 Effect of DCPH on Apoptosis in LPS‐Induced RAW264.7 Cells

**FIGURE 4:**
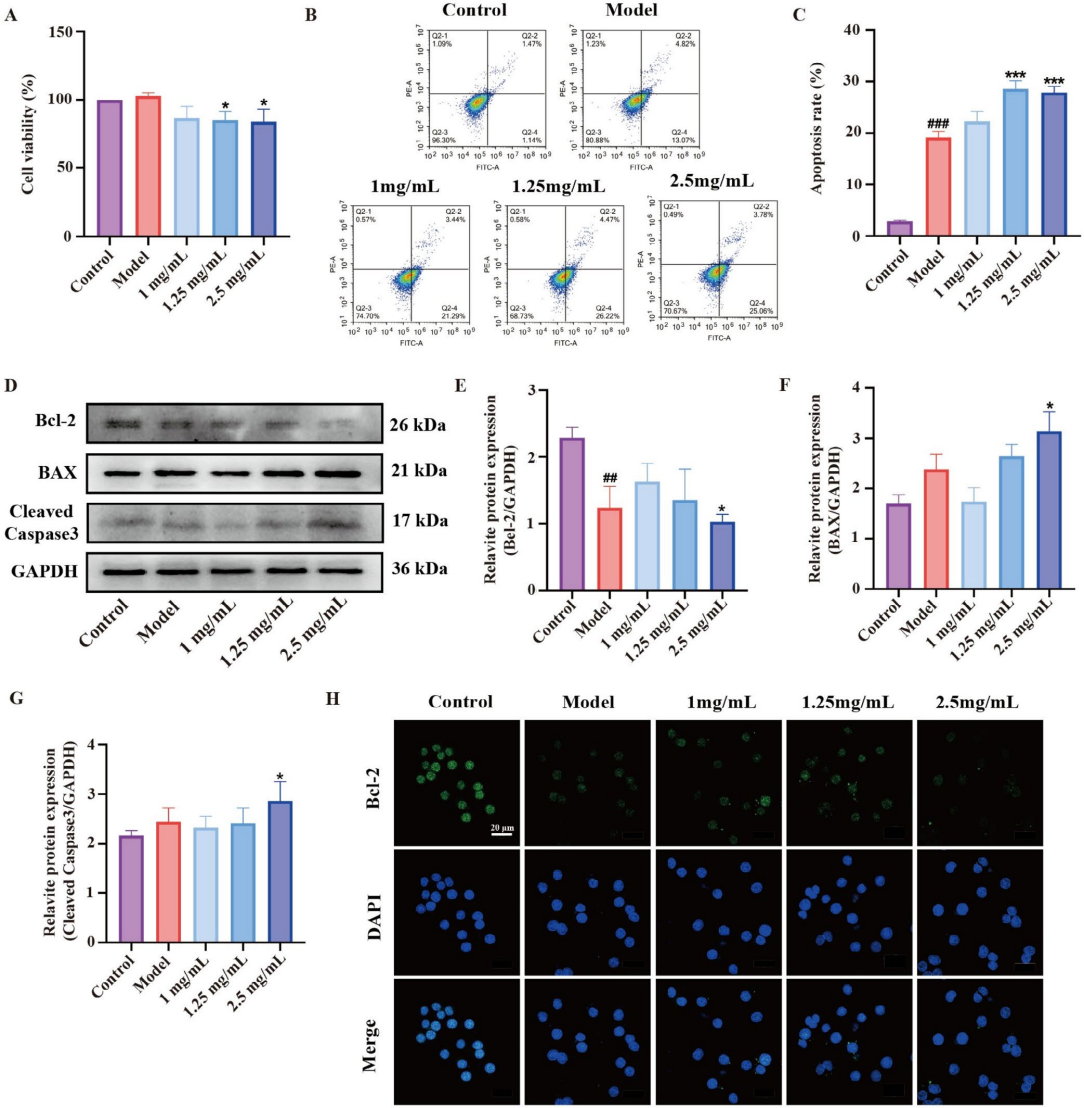
DCPH enhances apoptosis of RAW264.7 cells. (A) Effect of DCPH on the viability of RAW264.7 cells stimulated by LPS. (B) Apoptosis levels of RAW264.7 cells were determined by flow cytometry. (C) The cell apoptosis rate in (B). (D–G) The relative protein expression of BAX, BCL‐2, and cleaved caspase 3 in RAW264.7 cells were analyzed by western blotting. (H) The immunofluorescent representative images of BCL‐2 expression. Each experiment was repeated at least three times. Results are expressed as the mean ± standard deviation. ^###^ *p* < 0.001 compared to the control group; *p < 0.05, ***p < 0.001 compared to the model group. Ns, no significance.

Initially, we assessed the cell viability of DCPH in LPS‐induced RAW264.7 cells. The results indicated that LPS did not induce cytotoxicity in the cells; however, DCPH at concentrations of 1, 1.25, and 2.5 mg/mL exhibited a slight inhibitory effect on the viability of the LPS‐induced RAW264.7 cells (Figure 4A). Flow cytometry was employed to assess apoptosis levels in RAW264.7 cells following DCPH treatment. The results indicated a significant increase in apoptosis levels in RAW264.7 cells after DCPH treatment. Following a 12‐h LPS treatment, cells showed apoptosis levels of 17.89%. In contrast, apoptosis levels in the DCPH groups with doses of 1, 1.25, and 2.5 mg/mL were 24.73%, 30.69%, and 28.84%, respectively (Figure 4B,C). Additionally, DCPH upregulated the expression of the pro‐apoptotic protein Bax and Cleaved Caspase3, and downregulated the expression of the anti‐apoptotic protein Bcl‐2 compared to the model group (Figure 4D–G). These results were supported by immunofluorescence data showing a significant decrease in Bcl‐2 expression due to DCPH treatment (Figure 4H).

### 3.5 Network Pharmacological Analysis of DCPH With Oxidative Stress, Inflammation, Polarization and Apoptosis

**FIGURE 5:**
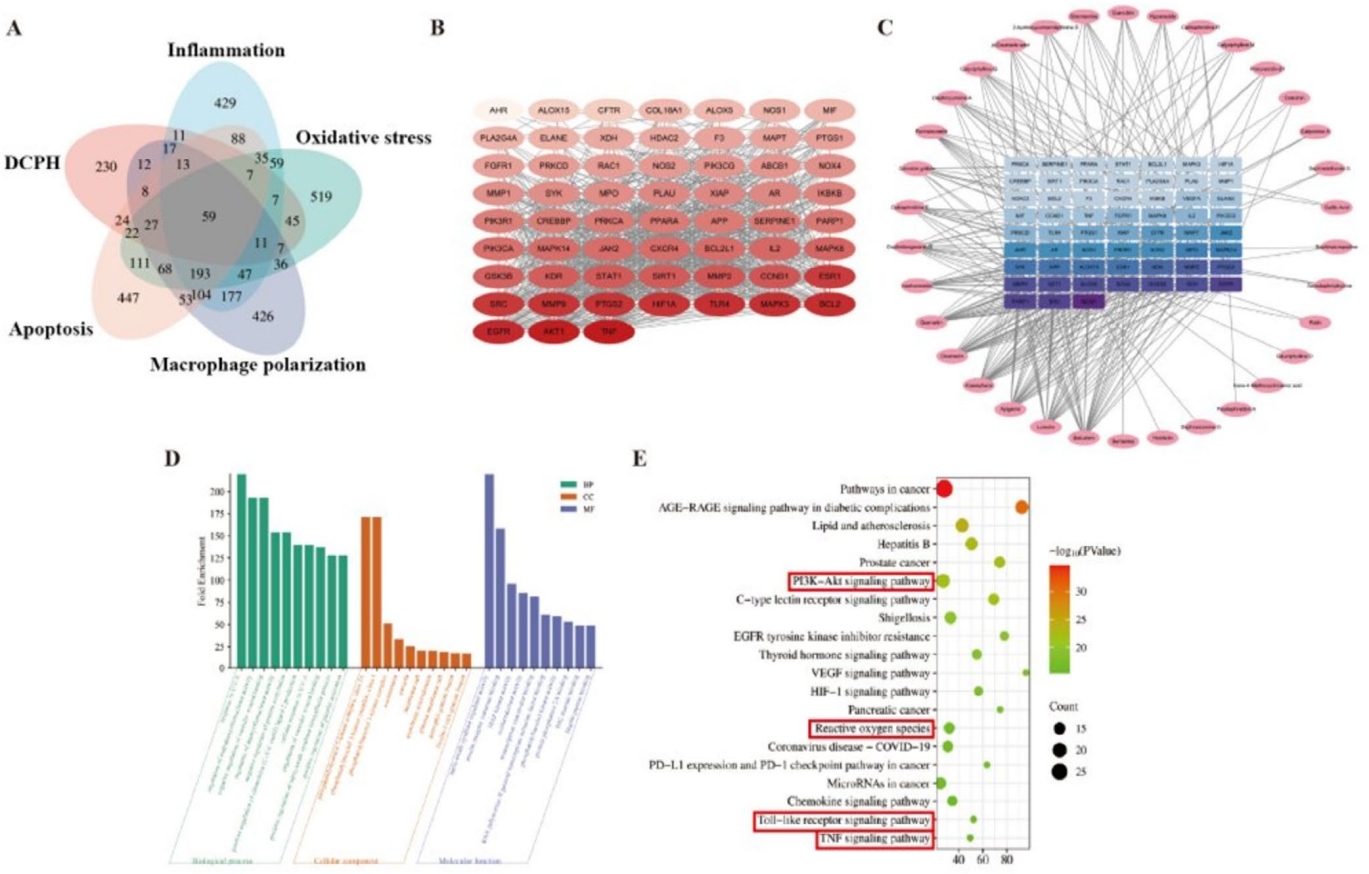
Predicted targets of DCPH. (A) Venn diagram of genes affected by DCPH and those involved in inflammation, oxidative stress, M1 polarization, and apoptosis. (B) Overlapping genes (59 genes). (C) Gene‐component network. (D) GO enrichment analysis of overlapping genes. (E) KEGG enrichment analysis of overlapping genes.

To elucidate the molecular mechanism of DCPH in oxidative stress, inflammation, M1 polarization, and apoptosis, relevant targets were compiled from GeneCards and Swiss Target Prediction. Analysis identified 59 genes closely associated with the anti‐oxidative stress, anti‐inflammatory, anti‐M1 polarization, and apoptosis‐modulating properties of DCPH (Figure 5A). A PPI network was constructed using these targets and visualized in Cytoscape 3.9.1 (Figure 5B). Key targets included TNF, AKT1, MAPK3, MMP9, EGFR, TLR4, BCL2, PARP1, PIK3CA, and PIK3R1. The relationship between DCPH's active compounds and the 59 common targets was illustrated in Figure 5C, showing major components such as Kaempferol, Quercetin, Diosmetin, Daphniyunnine A, Formononetin, Luteolin, Isorhamnetin, Sinomenine, Catechin, p‐Coumaric acid, Caluciphylline O, and Paxdaphnidne A. The study introduced these 59 key active intersecting targets into the Metascape platform for GO and KEGG enrichment analysis. GO analysis suggested involvement of inflammatory response, nitric‐oxide synthase regulator activity, and phosphatidylinositol kinase activity in the improvement of LPS‐induced RAW264.7 cells by DCPH (Figure 5D). Additionally, KEGG results in Figure 5E indicated potentially important pathways like the PI3K‐Akt pathway, Reactive oxygen species, Toll‐like receptor, and TNF signaling pathway. Specific compounds in DCPH were selected for molecular docking assays to evaluate their potential binding affinity with key receptors involved in the regulation of inflammation and autophagy, including TLR4, IKBKB, TNF, PIK3CA, PIK3R1, and BCL2 (Figure S1).

### 3.6 DCPH Exerts Anti‐Inflammatory Effects by Regulating the TLR4/NF‐κB Signaling Pathway

**FIGURE 6:**
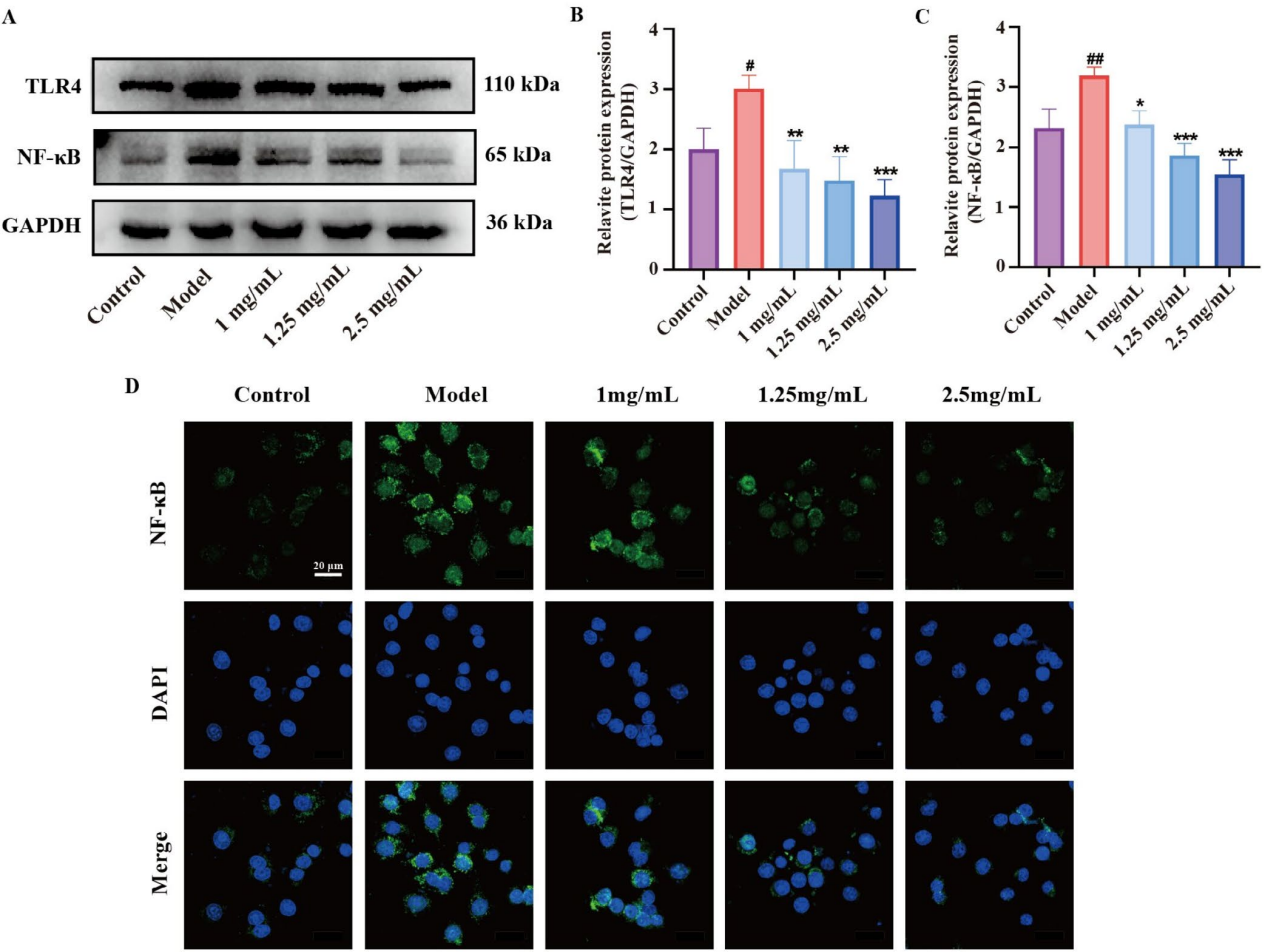
DCPH reduced the protein expression levels of TLR4 and NF‐κB. (A–C) The relative protein expression of TLR4 and NF‐κB in RAW264.7 cells were analyzed by western blotting. (D) The immunofluorescent representative images of NF‐κB expression. Each experiment was repeated at least three times. Results are expressed as the mean ± standard deviation. ^#^ *p* < 0.05, ^##^ *p* < 0.01 compared to the ontrol group; *p < 0.05, **p < 0.01, ***p < 0.001 compared to the codel group.

We further assessed the protein expression levels of TLR4 and NF‐κB in each cell group. Western blot results in Figure 6A show that TLR4 (Figure 6B) and NF‐κB (Figure 6C) protein levels were significantly higher in the model group compared to the control group. After DCPH pretreatment, there was a noticeable decrease in protein expression in a concentration‐dependent manner when compared to the model group. Immunofluorescence results also supported a significant decrease in NF‐κB protein expression with DCPH intervention (Figure 6D). These results indicate that the activation of the TLR4/NF‐κB pathway by LPS leads to an inflammatory response in RAW264.7 cells, and that this inflammation can be alleviated by inhibiting the TLR4/NF‐κB pathway post DCPH intervention, thereby demonstrating anti‐inflammatory properties.

### 3.7 DCPH Exhibits Anti‐Inflammatory and Protective Effects Against Gastric Mucosal Injury in Mice by Inhibiting the TLR4/NF‐κB/NLRP3 Pathway In Vivo

**FIGURE 7:**
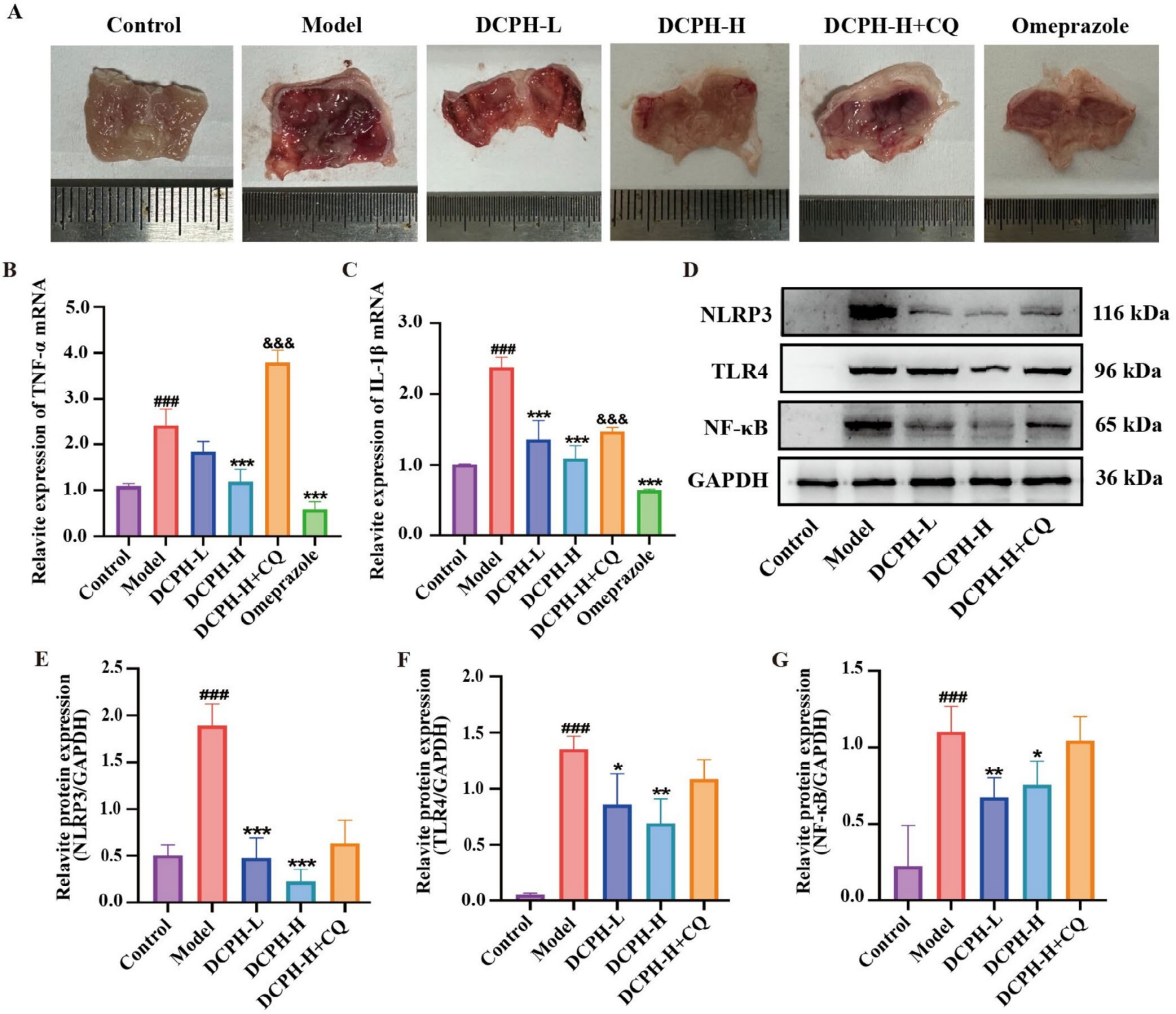
DCPH exhibits anti‐inflammatory and gastric mucosal protective effects in vivo. (A) Images of the protective effect of DCPH on ethanol‐induced gastric mucosal injury in mice. (B) The mRNA expression of proinflammatory cytokine TNF‐α. (C) The mRNA expression of proinflammatory cytokine IL‐1β. (D–G) The relative protein expressions of NLRP3, TLR4, and NF‐κB in gastric tissue were analyzed by western blotting. Each experiment was repeated at least three times. Results are expressed as the mean ± standard deviation. ^###^ *p* < 0.001 compared to the ontrol group; *p < 0.05, **p < 0.01, ***p < 0.001 compared to the cmodel group; ^&&&^ *p* < 0.001 compared to the DCPH‐H group. ns: O significance compared to the DCPH‐H group.

Our findings indicate that the area of injury in the DCPH‐L group, DCPH‐H group, and the Omeprazole group is significantly less when compared to the model group, showing a marked reduction in the ulcer index (%) of gastric mucosal. In contrast, the gastric mucosal injury remains considerable in the DCPH‐H + CQ group, which exhibits more severe damage than the DCPH‐H group, as illustrated in Figure 7A,B.

Concurrently, we assessed the mRNA expression of the inflammatory factors IL‐1β (Figure 7C) and TNF‐α (Figure 7D) in gastric tissue. The findings revealed that both DCPH and omeprazole significantly reduced the mRNA expression levels of IL‐1β and TNF‐α compared to the model group. In contrast, when comparing the DCPH‐H + CQ group to the DCPH‐H group, the mRNA expressions of IL‐1β and TNF‐α showed a significant increase.

In addition, we investigate the mechanism of DCPH in vivo. The results presented in Figure 7 indicate that the expressions of NLRP3 (Figure 7E), TLR4 (Figure 7F), and NF‐κB (Figure 7G) were significantly decreased in both the DCPH and omeprazole groups. However, when comparing the DCPH‐H group to the DCPH‐H + CQ group, the expressions of TLR4, NF‐κB, and NLRP3 increased, although this difference was not statistically significant. These results suggest that DCPH exerts an anti‐inflammatory effect in vivo by inhibiting the TLR4/NF‐κB/NLRP3 pathway, thereby protecting against gastric mucosal injury in mice.

### 3.8 DCPH Induces the Autophagy Level In Vivo and In Vitro

**FIGURE 8:**
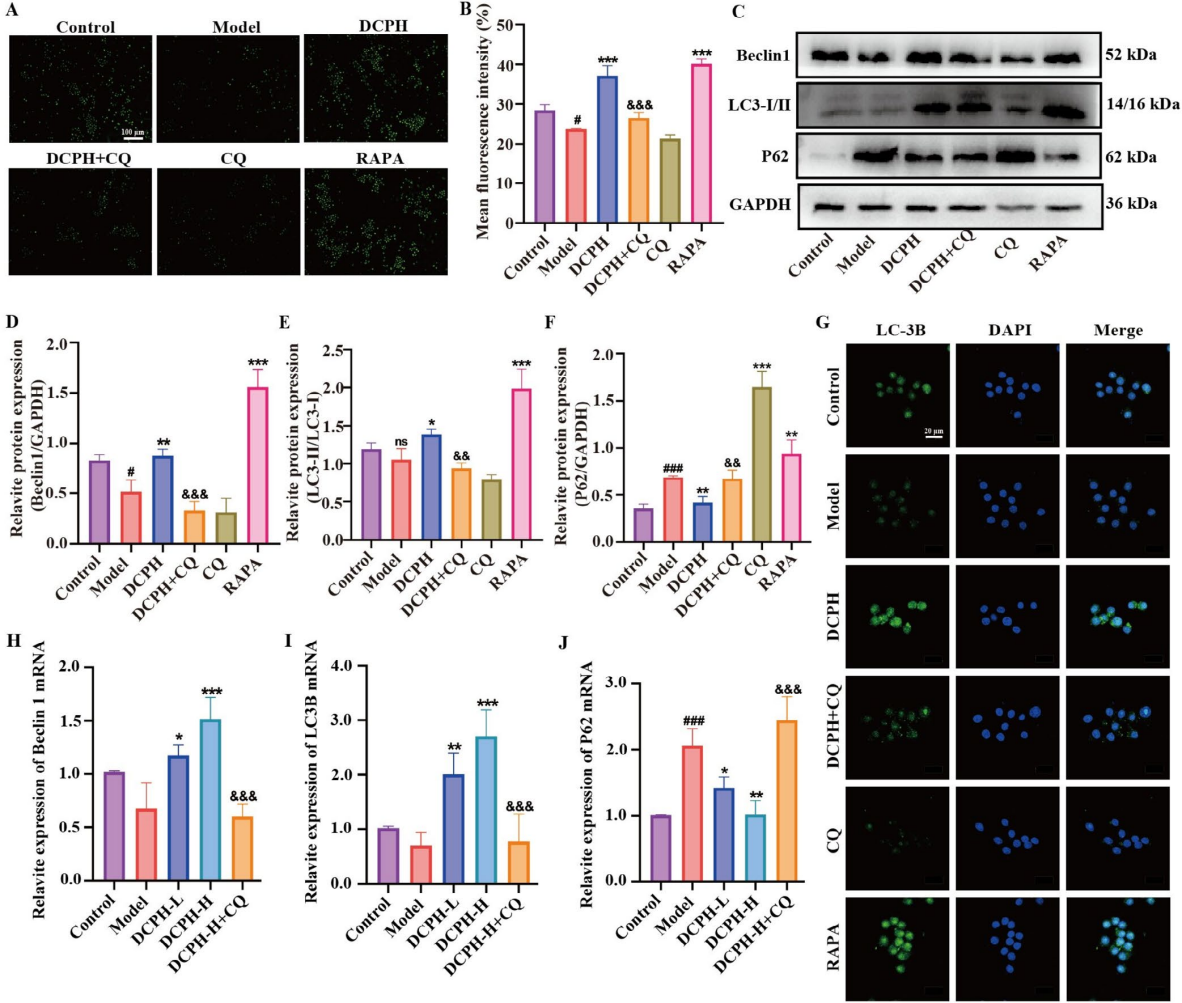
DCPH induces autophagy in vivo and in vitro. (A) The representative fluorescence images of autophagy in RAW264.7 cells observed by MDC method. (B) MDC mean fluorescence intensity in (A). (C–F) The relative protein expression of P62, Beclin 1, and LC3‐I/II in RAW264.7 cells was analyzed by western blotting. (G) The immunofluorescent representative images of LC3B expression. (H) The mRNA expression of Beclin1 in gastric tissue. (I) The mRNA expression of LC3B in gastric tissue. (J) The mRNA expression of P62 in gastric tissue. Each experiment was repeated at least three times. Results are expressed as the mean ± standard deviation. ^#^ *p* < 0.05, ^###^ *p* < 0.001 compared to the Control group; *p < 0.05, **p < 0.01, ***p < 0.001 compared to the Model group; ^&&^ *p* < 0.01, ^&&&^ *p* < 0.001 compared to the DCPH group. ns: O significance.

Our network pharmacology findings suggest that autophagy may play a role in the mechanism by which DCPH improves LPS‐induced inflammation in macrophages. To further investigate DCPH's impact on autophagy in RAW264.7 cells, we conducted the MDC assay to assess cellular autophagy and analyzed the expression levels of key autophagy proteins Beclin1, P62, and LC3‐II/I using western blot and immunofluorescence. Our results showed a significant decrease in cellular autophagy levels in the model group compared to the control group. In contrast, both DCPH and RAPA effectively induced autophagy in the cells when compared to the model group. Notably, the autophagy level in the DCPH+CQ group was significantly lower than that in the DCPH group (Figure 8A,B). Furthermore, in the model group, there was a noticeable decrease in Beclin1 expression, an important autophagy protein, and a significant increase in P62 expression. Conversely, treatment with DCPH and RAPA resulted in increased relative expression levels of Beclin1 and LC3‐II/I, along with a decrease in P62 expression. Additionally, compared to the DCPH group, the DCPH+CQ treatment significantly inhibited the expression of Beclin1 and LC3‐II/I while increasing P62 protein expression (Figure 8C–F). These results were supported by immunofluorescence analysis (Figure 8G). In vivo research results also indicate that DCPH can increase the expression of Beclin 1 and LC3B mRNA, while inhibiting the expression of P62 mRNA (Figure 8H–J). In conclusion, our data suggest that DCPH has a positive impact on inflammation in vivo and in vitro by promoting autophagy activation.

### 3.9 DCPH Regulates the TLR4/NF‐κB/NLRP3 Pathway Through the Autophagic, Thereby Inhibiting the Polarization of RAW264.7 Cells to the M1 Type

**FIGURE 9:**
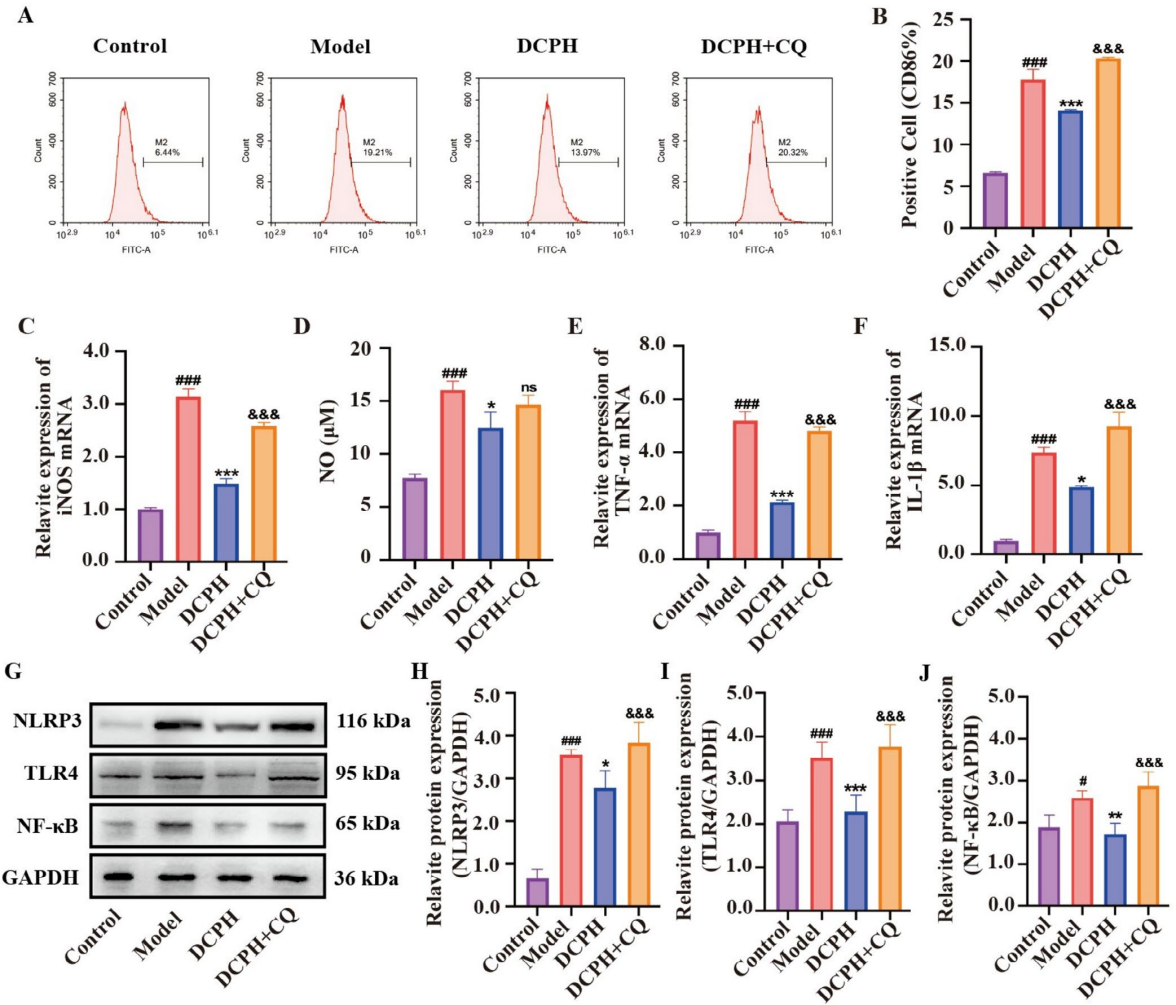
DCPH inhibited TLR4/NF‐κB/NLRP3 signaling pathway and M1 polarization through autophagy. (A) CD86 expression in RAW264.7 cells was measured by flow cytometry. (B) Percentage of CD86‐positive cells in (A). (C) The mRNA expression of *iNOS*. (D) The release of NO. (E) The mRNA expression of TNF‐α. (F) The mRNA expression of IL‐1β. (G–J) The relative protein expression of NLRP3, TLR4, and NF‐κB in RAW264.7 cells was analyzed by western blotting. Each experiment was repeated at least three times. Results are expressed as the mean ± standard deviation. ^#^ *p* < 0.05, ^###^ *p* < 0.001 compared to the control group; *p < 0.05, **p < 0.01, ***p < 0.001 compared to the code group; ^&&&^ *p* < 0.001 compared to the mFLCWK group. ns, no significance.

Further investigations delved deeper into the process by which DCPH activates autophagy to alleviate cell inflammation. The impact of DCPH on RAW264.7 cells was validated through the assessment of CD86 and iNOS expression, NO levels, as well as TNF‐α and IL‐1β inflammatory factors expression. Our findings demonstrated that exposure to LPS resulted in increased CD86 expression (Figure 9A,B), upregulated iNOS mRNA expression (Figure 9C), elevated NO release (Figure 9D), and enhanced TNF‐α (Figure 9E) and IL‐1β (Figure 9F) mRNA expression, ultimately leading to cellular inflammation. Interestingly, similar effects were observed in the CQ group. However, both DCPH effectively reversed this process by reducing CD86 expression, suppressing iNOS mRNA expression and NO release, and diminishing TNF‐α and IL‐1β mRNA expression, thereby mitigating cellular inflammation. These findings suggest that DCPH may mitigate M1 polarization of cells by activating autophagy.

To delve deeper into the molecular mechanisms of DCPH in regulating macrophage polarization through autophagy, we conducted western blot analysis to assess the expression of proteins associated with the TLR4/NF‐κB/NLRP3 signaling pathway. The results depicted in Figure 9G indicate that treatment with DCPH led to a significant decrease in the protein levels of NLRP3 (Figure 9H), TLR4 (Figure 9I), and NF‐κB (Figure 9J). Conversely, the addition of CQ reversed the inhibitory impact of DCPH on these proteins. These results suggest that DCPH exerts its inhibitory effects on the TLR4/NF‐κB/NLRP3 pathway by promoting autophagy, thereby mitigating M1 polarization in cells and reducing inflammation.

## 4 Discussion

DCPH, a Chinese drug pair, is commonly used to treat gastric ulcers, acute and chronic gastroenteritis in clinical settings. But due to the complexity and holistic nature of TCM, its mechanism of action remains unclear. In this study, the UPLC‐MS/MS was used to identify the main flavonoid components in DCPH, and we induced inflammation in RAW264.7 cells using LPS and observed that DCPH exhibited various protective effects on these cells, including anti‐oxidative, anti‐inflammatory, inducing apoptosis of damaged cells, and anti‐M1 polarization properties. Furthermore, network pharmacological analysis and further mechanism exploration were conducted. The findings suggest that autophagy and the TLR4/NF‐κB/NLRP3 signaling pathway may play a role in the anti‐inflammatory effects of DCPH.

Oxidative stress is characterized by either excessive production or insufficient removal of highly active molecules in the body, leading to tissue damage. This stress can result in an increase in ROS, a key feature of the macrophage inflammatory response (Ren et al. 2024; Yu et al. 2024). The findings of this study indicate that DCPH can effectively lower ROS levels, reduce the expression of *TNF‐α* and *IL‐1β*, inhibit M1 polarization and promote M2 polarization induced by LPS. Studies have indicated that ROS can trigger the M1 polarization of macrophages, prompting the release of inflammatory factors, chemokines, and adhesion molecules like *TNF*, *IL‐1β*, *iNOS*, and NO, thereby initiating an inflammatory cascade (Tsai et al. 2024; Liu et al. 2025; Hu et al. 2023; Bahiraii et al. 2024). Furthermore, research has demonstrated that combating oxidative stress can suppress the inflammatory response (Weinberg Sibony et al. 2024).

Network pharmacology has become a popular method in recent years for studying the interactions between active compounds, proteins, and diseases. Our results from network pharmacology studies suggest that the reactive oxygen species, PI3K‐Akt, Toll‐like receptor and TNF signaling pathway may play a role in the improvement of macrophage injury by DCPH. Our findings have demonstrated that DCPH blocks the TLR4/NF‐κB/NLRP3 signaling pathway to reduce ROS levels and suppress macrophage M1 polarization, mitigating cellular inflammation. Previous research suggested that the TLR4/NF‐κB/NLRP3 pathway, a well‐known inflammatory signaling pathway, is involved in regulating macrophage polarization (Alanazi et al. 2025). TLR4 is a pattern recognition receptor, which can sense the invasion of pathogenic microorganisms and tissue damage in immune cells (such as macrophages) (Fisch et al. 2024). Activation of TLR4 by specific external substances (such as LPS) leads to the recruitment of the Myd88 receptor, triggering IκB phosphorylation, initiating the NF‐κB (Kim et al. 2023; S. Zhang et al. 2024). This process promotes the formation of the NLRP3 inflammasome, and finally secretes IL‐1β, IL‐18 and TNF‐α, thus aggravating the inflammatory reaction (Khan et al. 2025; Blevins et al. 2022). It has also been found that LPS stimulates TLR4 receptors to induce a large number of ROS production, which in turn activates NF‐κB and NLRP3, and finally amplifies the inflammatory response (Hu et al. 2024; Sharma et al. 2024). Research indicates that a variety of plant medicines and Chinese patent medicines can effectively modulate this signaling pathway to improve inflammatory response. For instance, studies have shown that the main active components of Taxus chinensis fruit extract, such as gallocatechin, catechin, kaempferol, quercetin, rutin, naringin, and apigenin, et al., have strong affinity with TLR4, suggesting that the molecular basis of their anti‐inflammatory effects may be through hydrogen bonding, aromatic cation‐π interaction and hydrophobic interaction (Meimei et al. 2025). These compounds are also the main active components of DCPH. However, the specific molecular mechanism by which DCPH inhibits the TLR4/NF‐κB/NLRP3 pathway requires further investigation.

An additional intriguing discovery revealed that the rise in apoptosis noted in the DCPH group exceeded that of the LPS group, contradicting the expected protective effect of DCPH on RAW264.7 cells. Previous studies have shown that macrophage apoptosis in sepsis‐induced acute kidney injury (AKI) reduces the release of pro‐inflammatory cytokines and ROS. By eliminating damaged macrophages, apoptotic processes limit renal tissue damage and preserve microcirculation homeostasis, thereby protecting adjacent normal cells from oxidative stress and inflammatory cascades (Li et al. 2021). Furthermore, inhibition of the PI3K/AKT pathway may lead to increased levels of apoptosis, which has an anti‐inflammatory effect (Chen et al. 2021; Rao et al. 2022; Sreenivasulu et al. 2020). The results emphasize that high levels of apoptosis help to clear damaged cells and limit the intensity and duration of inflammatory responses, which is a key mechanism to reduce secondary damage caused by macrophage dysfunction.

Autophagy is a highly conserved cellular degradation pathway that utilizes lysosomes to break down damaged organelles, macromolecules, and other substances. The resulting amino acids and small molecules are then recycled, promoting the circulation of intracellular substances and maintaining internal balance (Vargas et al. 2023; Yamamoto et al. 2023). Key proteins in autophagy include LC3 and Beclin 1, with the LC3‐II/I ratio serving as a crucial indicator of autophagy levels, and Beclin 1 acting as a marker for the initiation of autophagic flow (Prerna and Dubey 2022; Piletic et al. 2023). The protein P62, a product of autophagic degradation, tends to accumulate when autophagy is impaired or autophagic flow is obstructed, making it a valuable indicator of autophagy function (Klionsky et al. 2021). Autophagy serves as a crucial survival mechanism during stress conditions, maintaining intracellular homeostasis while also influencing inflammation and immune system regulation, particularly in relation to macrophage polarization. In this research, DCPH was found to inhibit the TLR4/NF‐κB/NLRP3 signaling pathway by inducing autophagy in vitro, consequently suppressing cellular M1 polarization and reducing inflammation. Various pathways such as NF‐κB, AMPK/mTOR, NLRP3 inflammasome, and PI3K/Akt are involved in the regulation of autophagy towards macrophage polarization (Chen, Guan, and Ren 2020; Vitaliti et al. 2025; Liu et al. 2021; Kumari et al. 2024). Additionally, the impact of the PI3K/Akt/mTOR signaling pathway on autophagy regulation has garnered considerable attention. Research indicates that titanium particles induce apoptosis in macrophages by enhancing autophagy through the PI3K/Akt pathway, resulting in immune dysfunction and decreased secretion of IL‐1β, IL‐6, and TNF‐α (Xian et al. 2020). Therefore, it is hypothesized that DCPH may induce macrophage autophagy via the PI3K/Akt/mTOR pathway, inhibiting M1 polarization, although further investigation is Warranted.

## 5 Conclusion

In summary, we identified active ingredients in DCPH with anti‐gastric injury activity via UPLC‐MS/MS and bioinformatics, and performed molecular docking against key targets (TLR4, NF‐κB, NLRP3, BCL2). Network pharmacology suggested that its mechanism involves the suppression of oxidative stress, inflammation, apoptosis, and M1 polarization, with autophagy and TLR4/NF‐κB/NLRP3 pathway serving as crucial mediators. In vivo and in vitro experiments confirmed DCPH ameliorates mouse gastric mucosal injury by inhibiting this pathway, with efficacy comparable to omeprazole. Notably, DCPH suppresses the pathway and M1 polarization by promoting autophagy, thereby reducing cell inflammation. These findings offer a novel therapeutic strategy for managing gastritis and gastric ulcers.

## Author Contributions

**Yunyun Zhi:** writing – original draft, data curation, formal analysis. **Keli Zhou:** writing – original draft, software, formal analysis. **Yonghui Li:** methodology, investigation, supervision. **Xueqing Huang:** visualization, software, data curation. **Shouzhong Ren:** writing – review and editing, funding acquisition, methodology. **Ye Zhu:** writing – review and editing, project administration, resources. **Yiqiang Xie:** writing – review and editing, investigation, conceptualization, validation.

## Funding

This work was supported by the National Natural Science Foundation of China (82260920, 82474456), Academic Enhancement Support Program of Hainan Medical University (XSTS2025173), Undergraduate Research and Innovation Training Program of Hainan Medical University (RZ2500002208).

## Conflicts of Interest

The authors declare no conflicts of interest.

## Supporting information


**Figure S1:** Molecular docking of DCPH‐derived compounds. Specific compounds derived from DCPH were selected for molecular docking assays to evaluate their potential binding affinity with key receptors involved in the regulation of inflammation and autophagy.

## Data Availability

The data presented in this study are available in the article.
