# Supplementary figures and images for: The Herbal Pair Daphniphyllum calycinum– Polygonum hydropiper Alleviates Gastric Mucosal Injury via Regulating Autophagy and Targeting the TLR4/NF‐κB/NLRP3 Pathway
Source: Food Sci Nutr. 2026 Apr 12;14(4):e71768. doi: 10.1002/fsn3.71768 (PMC13071168; doi:10.1002/fsn3.71768)

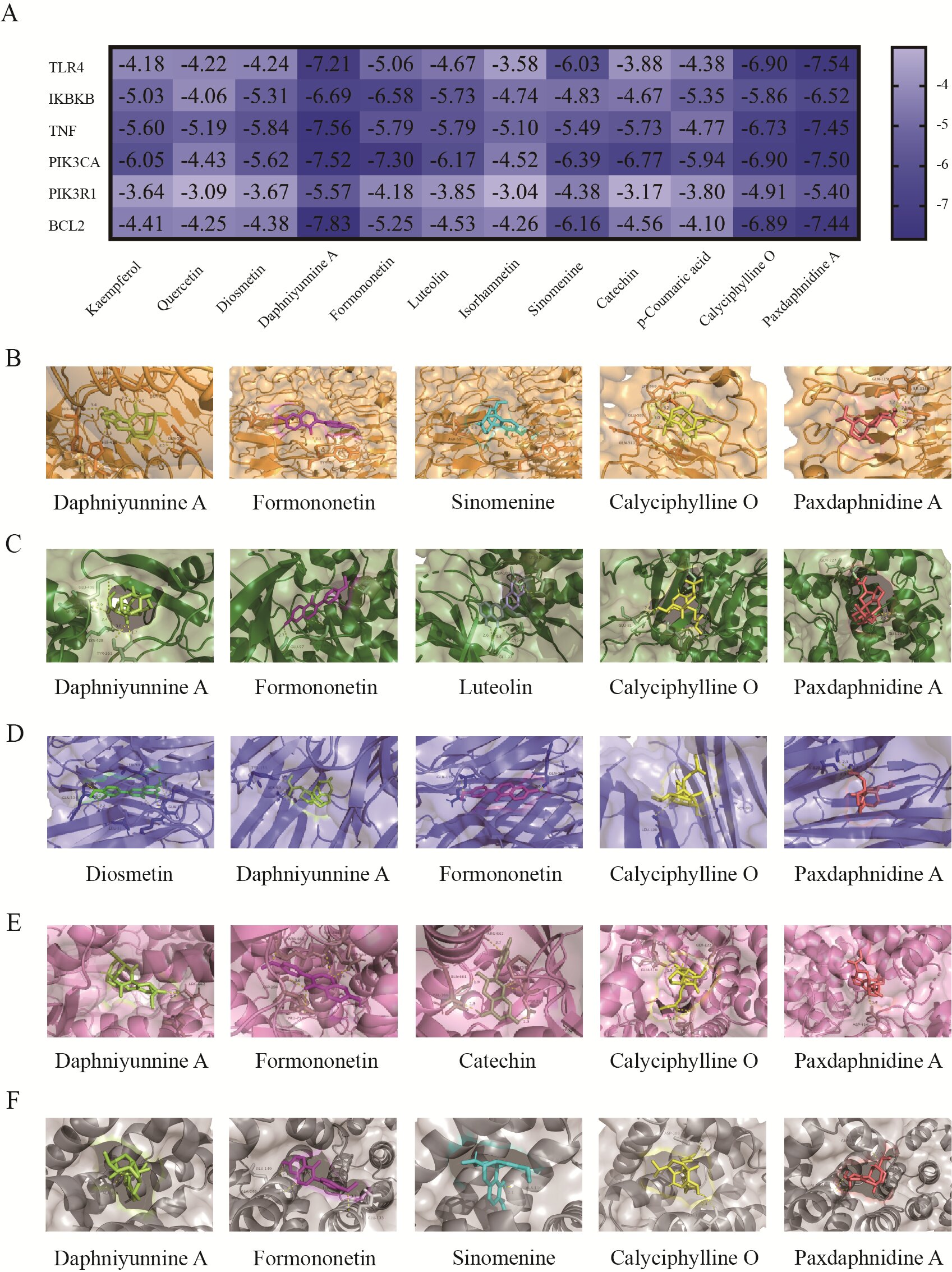

Supplement: Supplementary file 1 — Figure S1: Molecular docking of DCPH‐derived compounds. Specific compounds derived from DCPH were selected for molecular docking assays to evaluate their potential binding affinity with key receptors involved in the regulation of inflammation and autophagy. [file FSN3-14-e71768-s001.jpg]
